# Measurement of the Red Blood Cell Distribution Width Improves the Risk Prediction in Cardiac Resynchronization Therapy

**DOI:** 10.1155/2016/7304538

**Published:** 2016-01-19

**Authors:** András Mihály Boros, Péter Perge, Zsigmond Jenei, Júlia Karády, Endre Zima, Levente Molnár, Dávid Becker, László Gellér, Zoltán Prohászka, Béla Merkely, Gábor Széplaki

**Affiliations:** ^1^Heart and Vascular Center, Semmelweis University, Városmajor Utca 68, Budapest 1122, Hungary; ^2^Third Department of Internal Medicine, Semmelweis University, Budapest, Hungary

## Abstract

*Objectives*. Increases in red blood cell distribution width (RDW) and NT-proBNP (N-terminal pro-B-type natriuretic peptide) predict the mortality of chronic heart failure patients undergoing cardiac resynchronization therapy (CRT). It was hypothesized that RDW is independent of and possibly even superior to NT-proBNP from the aspect of long-term mortality prediction.* Design*. The blood counts and serum NT-proBNP levels of 134 patients undergoing CRT were measured. Multivariable Cox regression models were applied and reclassification analyses were performed.* Results*. After separate adjustment to the basic model of left bundle branch block, beta blocker therapy, and serum creatinine, both the RDW > 13.35% and NT-proBNP > 1975 pg/mL predicted the 5-year mortality (*n* = 57). In the final model including all variables, the RDW [HR = 2.49 (1.27–4.86); *p* = 0.008] remained a significant predictor, whereas the NT-proBNP [HR = 1.18 (0.93–3.51); *p* = 0.07] lost its predictive value. On addition of the RDW measurement, a 64% net reclassification improvement and a 3% integrated discrimination improvement were achieved over the NT-proBNP-adjusted basic model.* Conclusions*. Increased RDW levels accurately predict the long-term mortality of CRT patients independently of NT-proBNP. Reclassification analysis revealed that the RDW improves the risk stratification and could enhance the optimal patient selection for CRT.

## 1. Introduction

Chronic heart failure is characterized by reduced cardiac contractility and an impaired stroke volume. These changes activate the sympathetic nervous system and cause a volume redistribution and overload. The intravascular fluid excess elevates the concentrations of circulating natriuretic peptides, such as the N-terminal pro-B-type natriuretic peptide (NT-proBNP), to compensate the hemodynamic alterations. NT-proBNP is therefore regarded as a disease-specific biomarker and is routinely used in diagnosis, therapy guidance, and prognosis estimation for chronic heart failure patients [1]. Nevertheless, NT-proBNP mirrors only the actual volume homeostasis, which is a rather simplified aspect of the multifaceted pathophysiology.

The red blood cell distribution width (RDW) is a measure of the variation of the erythrocyte size, and an increased RDW is also linked to a poor prognosis in cardiovascular diseases, such as coronary artery disease [2], acute myocardial infarction [3], stroke [4], and chronic heart failure [5–8]. Interestingly, diabetes mellitus [9], liver diseases [10], and renal dysfunctions [11] may likewise lead to RDW elevations, the available evidence therefore suggesting that an increased RDW reflects the presence and extent of a systemic injury caused by the underlying disease.

Cardiac resynchronization therapy (CRT) is widely used to treat chronic heart failure patients with electromechanical dyssynchrony and a deteriorating functional status [12]. It is well established that CRT improves the cardiac function, exerts reverse remodeling, and decreases the need for hospitalization, though the mortality remains rather high [13].

The aim of this study was to measure the RDW and NT-proBNP levels before CRT, for we hypothesized that these biomarkers, either alone or in combination, can predict the long-term mortality following CRT. Indeed, it has recently been demonstrated that increasing RDW levels predict mortality before CRT, although it remained unclear whether the NT-proBNP data would affect the strength of the prediction [14–16]. Accordingly, we performed multivariable survival and reclassification analyses in order to determine the value of the NT-proBNP and the RDW in the long-term prognosis prediction.

## 2. Material and Methods

### 2.1. Study Population and Study Design

A cohort of 141 consecutive patients referred to our Heart and Vascular Center between September 2009 and December 2010 was included in this prospective single-center observational study. We aimed to evaluate the prognostic impact of routine and novel laboratory biomarkers in chronic heart failure patients with CRT. We have shown previously the predictive role of white blood cells in CRT [17]. The present analysis focuses on the impact of the RDW in the same patient population.

CRT was indicated by medically refractory chronic heart failure in accordance with the current guidelines [18]. Briefly, the cohort comprised chronic heart failure patients on optimal medical treatment with an impaired New York Heart Association (NYHA) functional status (II–IV), a severely reduced (<35%) left ventricular ejection fraction (LVEF), and a wide QRS in the ECG (>120 msec). Four patients were excluded because of history of autoimmune disease, hematologic disease, an acute or chronic inflammatory disease, or a malignancy. Experienced electrophysiologists performed the CRT by insertion of a left ventricular lead into the coronary sinus and a right ventricular lead in a septal position. Routine laboratory tests, clinical examinations, and ECG and echocardiographic measurements were carried out at baseline and 6 months later. Cardiologists analyzed the echocardiographic parameters in a blinded fashion in order to calculate the LVEF with Simpson's biplane method and the left ventricle volumes with the Teicholz method.

Prior to the enrollment, the local Ethics Committee of the Semmelweis University had approved the protocol, which was in accordance with the Helsinki Declaration, and all of the patients provided their written informed consent.

Complete laboratory and echocardiographic data on a total of 134 patients were included in the final analysis. The all-cause 5-year mortality was taken as the end-point of the study. Echocardiographic reverse remodeling was defined as an improvement of at least 15% in the left ventricular end-systolic volume 6 months after CRT implantation without death.

### 2.2. Laboratory Measurements

Study personnel collected ethylenediaminetetraacetic acid and citrate-treated serum samples for routine biochemical measurements before CRT and 6 months later. Red blood cell morphology parameters were recorded with the Symex XS-1000i (Kobe, Japan) system by means of a direct current detection method and hydrodynamic focusing technology. Levels of NT-proBNP were measured with an electrochemiluminescence Cobas e 411 analyzer (Mannheim, Germany), using Roche Elecsys NT-proBNP II kits (Cat. number: 04842464190, Mannheim, Germany).

### 2.3. Statistical Analysis

A two-tailed *p* value of < 0.05 was considered statistically significant in all cases. The statistical analysis was carried out by using IBM SPSS 22 (Apache Software Foundation, USA), Graphpad Prism 6.03 (GraphPad Softwares Inc, USA), and PASS 2008 (NCSS, USA) the open source R software (R version 3.1.2 with PredictABEL and pROC packages).

The data were expressed as medians with interquartile ranges or as percentages with event numbers and the Mann-Whitney test or the chi squared test was applied for the comparison of two groups, as appropriate. Univariate Cox regression analysis was used to determine the 5-year mortality predictors. The continuous variables were standardized by a one standard deviation (SD) increase (*Z* transformation). Receiver operating characteristic analysis was used and the continuous variables were dichotomized in such a way as to obtain identical sensitivity values, and the Kaplan-Meier curves were then compared by using log-rank tests. In the multivariable Cox regression models, the basic model included variables with *p* < 0.05 values of the univariate analysis, and further adjusted models were built in a forward stepwise manner [19]. A second multivariable model was performed with factors that gave *p* < 0.15 in the univariate analysis in order to present a broader view of the prediction. Power analysis was performed, and the validity of the results was checked and the performance of the models was characterized via the Brier score and Nagelkerke's *R* ^2^. Finally, *C*-statistics with the DeLong test was carried out to display the overall areas under the curves of the models and for reclassification analyses, including net reclassification improvement (NRI) and integrated discrimination improvement (IDI) [20].

## 3. Results

### 3.1. Baseline Characteristics of the Study Population

The median age of the 134 patients was 67 years, 82% of them were male, 57% had ischemic heart failure, and 82% exhibited a left bundle branch block (LBBB) in the ECG, as shown in Table 1.

### 3.2. Changes in Echocardiographic and Laboratory Parameters

**Figure 1:**
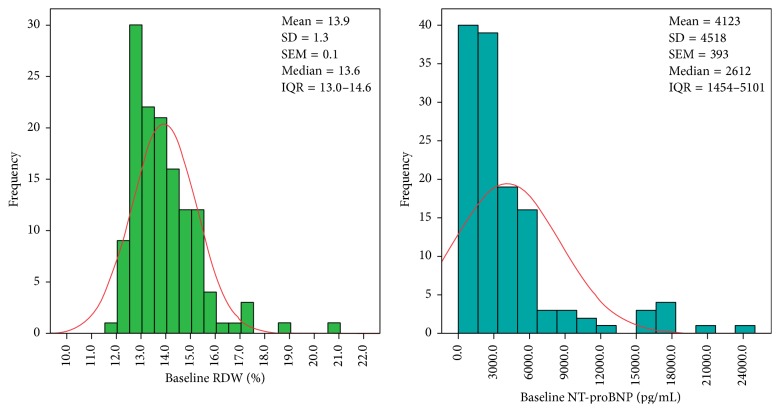
Histogram of baseline RDW and NT-proBNP. Both the RDW and NT-proBNP differ from the red line of normal distribution. RDW = red blood cell distribution width; NT-proBNP = N-terminal pro-B-type natriuretic peptide; SD = standard deviation; SEM = standard error of the mean; IQR = interquartile range (25% percentile–75% percentile).

The patients experienced echocardiographic reverse remodeling 6 months after CRT implantation. LVEF increased [28% (23–33) versus 37% (30–41); *p* < 0.0001], while LVESV [210 (153–276) versus 167 (115–242) mL; *p* < 0.0001] and LVEDV [303 (250–361) versus 259 (202–324) mL; *p* < 0.0001] decreased statistically significantly. The RDW, hematocrit, and creatinine remained statistically unaltered [13.6% (13.0–14.6) versus 13.4% (13.0–14.2); *p* = 0.56 and 42.3% (38.2–45.0) versus 41.4% (38.3–43.4); *p* = 0.06, and 109 (79–134) versus 96 (80–130) *μ*mol/L; *p* = 0.73, resp.], whereas NT-proBNP decreased significantly [2612 (1454–5101) versus 1626 (725–3300) pg/mL; *p* < 0.0001].  Figure 1 illustrates the baseline RDW and NT-proBNP distributions.

A total of 123 patients were analyzed for reverse remodeling. The responders to CRT (*n* = 61, 50%) were younger and presented with higher left ventricular volumes, a better NYHA functional status, and more frequent MRI usage, as described in Table 2. Increasing baseline levels of RDW [odds ratio (OR) = 1.52 (1.01–2.29); *p* = 0.04, per 1 SD increase], NT-proBNP [OR = 2.00 (1.19–3.38); *p* = 0.009] and creatinine [OR = 1.56 (1.04–2.34); *p* = 0.02] predicted the lack of reverse remodeling (*n* = 62, 50%) in univariate logistic regression analysis. Hematocrit concentrations were not associated with reverse remodeling [OR = 0.70 (0.49–1.02); *p* = 0.06]. In the multivariable analysis involving the significant factors (*p* < 0.05) of the univariate models, only NT-proBNP [OR = 2.67 (1.06–6.69); *p* = 0.03] remained statistically significant laboratory predictor of a nonresponse [creatinine OR = 1.46 (0.90–2.41); *p* = 0.12 and RDW OR = 1.01 (0.62–0.63); *p* = 0.95].

### 3.3. Survival Analysis

Up to a median follow-up of 1799 days (maximum 2181 days), 57 (42%) patients died (Table 1). Those patients survived longer, who displayed LBBB morphology in the ECG or were on beta-blocker therapy. Increasing baseline levels of RDW [hazard ratio (HR) = 1.48 (1.25–1.75), *p* < 0.0001; per 1 SD increase], NT-proBNP [HR = 1.43 (1.19–1.73); *p* < 0.0001], and serum creatinine [HR = 1.42 (1.18–1.71); *p* < 0.0001] worsened the long-term survival chances. Elevated hematocrit fractions improved the survival [HR = 0.70 (0.53–0.92); *p* = 0.01]. Older age (*p* = 0.08), male gender (*p* = 0.09), poor NYHA class, and diabetes mellitus (*p* = 0.12) tended to be associated with an adverse outcome.

Receiver operating characteristic analysis was performed to obtain optimal cut-off values. Each laboratory parameter was dichotomized so as to reach a sensitivity of 79% (66–88) in mortality prediction, which we considered clinically meaningful. In this way, the individual specificity values were different, but the sensitivity was the same in all cases, making the cut-off selection more objective and comparable.

**Figure 2:**
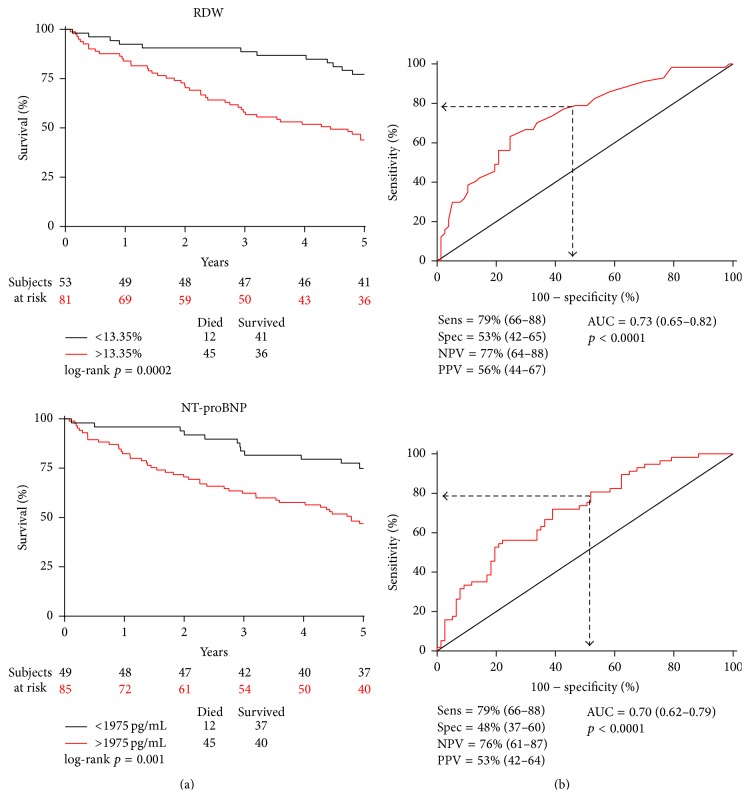
Increased RDW and NT-proBNP levels predict the 5-year mortality of chronic heart failure patients before CRT. We performed receiver operating characteristic analysis (a) and dichotomized the continuous variables so as to obtain the same sensitivity values. The Kaplan-Meier survival curves were tested by the log-rank test (b). CRT = cardiac resynchronization therapy; RDW = red blood cell distribution width; NT-proBNP = N-terminal pro-B-type natriuretic peptide; AUC = area under the curve; Sens = sensitivity; Spec = specificity; NPV = negative predictive value; PPV = positive predictive value.

Patients before CRT were subject to up to a 3-fold increased 5-year mortality risk, with RDW levels > l3.35% [HR = 3.20 (1.69–6.06), *p* = 0.0002; specificity = 53% (42–65)], NT-proBNP levels > 1975 pg/mL [HR = 2.71 (1.43–5.14), *p* = 0.001; specificity = 48% (37–60)] and serum creatinine > 88.5 *μ*mol/L [HR = 2.80 (1.45–5.42), *p* = 0.001; specificity = 47% (35–59)], as shown in Figure 2. A hematocrit < 44% [HR = 1.59 (0.84–3.00), *p* = 0.15; specificity = 34% (24–45)] did not predict mortality statistically significantly.

Patients with elevated RDW levels before CRT implantation were in a worse NYHA functional class, had less LBBB pattern in the ECG with wider QRS, and used less angiotensin convertase inhibitor therapy, as shown in Table 3. They also tended to be older and to have diabetes more frequently. These patients exhibited significantly elevated NT-proBNP and creatinine concentrations but statistically indifferent hematocrit levels.

We performed multivariable Cox regression analyses and set up a basic prediction model with significant (*p* < 0.05) clinical (LBBB and beta-blocker therapy) and laboratory parameters (serum creatinine > 88.5 *μ*mol/L). After separate adjustment to the basic model, both the RDW > 13.35% [HR = 2.81 (1.45–5.44); *p* = 0.002] and NT-proBNP > 1975 pg/mL [HR = 2.19 (1.13–4.23); *p* = 0.01] predicted the 5-year mortality (Table 4). In the final model including all variables, the RDW remained significant [HR = 2.49 (1.27–4.86); *p* = 0.008], whereas the NT-proBNP lost its predictive value [HR = 1.18 (0.93–3.51); *p* = 0.07].

We additionally implemented a multivariable model 2, in which the basic model included extended clinical (age, male gender, NYHA class III/IV, LBBB, beta-blocker therapy, and diabetes mellitus) and laboratory (serum creatinine > 88.5 *μ*mol/L) factors with *p* < 0.15 from the univariate analysis (Table 5). Similarly, after separate adjustment to the basic model, both the RDW > 13.35% [HR = 2.07 (1.38–5.29); *p* = 0.004] and NT-proBNP > 1975 pg/mL [HR = 2.21 (1.13–4.33); *p* = 0.02] predicted the 5-year mortality. In the final model including all variables, the RDW remained significant [HR = 2.42 (1.22–4.76); *p* = 0.01], whereas the NT-proBNP lost its predictive value [HR = 1.88 (0.95–3.69); *p* = 0.06].

### 3.4. Validation, Performance, and Reclassification

**Figure 3:**
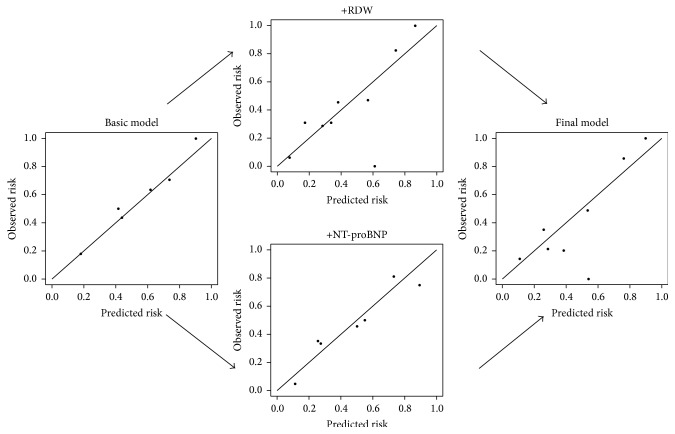
Calibration plots of the prediction models. The observed and predicted risks converge to the line of perfect prediction, suggesting the good calibration of the models. RDW = red blood cell distribution width; NT-proBNP = N-terminal pro-B-type natriuretic peptide.

The power analysis demonstrated that the RDW had a predictive power of 84% in the population of 134 patients to detect those 45 patients who died with an elevated RDW. The predicted and observed risks, assessed with the Hosmer-Lemeshow test, were similar throughout the analyses, as an indication of good calibration and confirming the validity (Figure 3, Tables 4 and 5). The Brier Score and Nagelkerke  *R* ^2^ measure the accuracy of survival predictions. The changes (a decreasing Brier score and an increasing Nagelkerke *R* ^2^) in these scores suggest an improved prediction (Tables 4 and 5).

**Figure 4:**
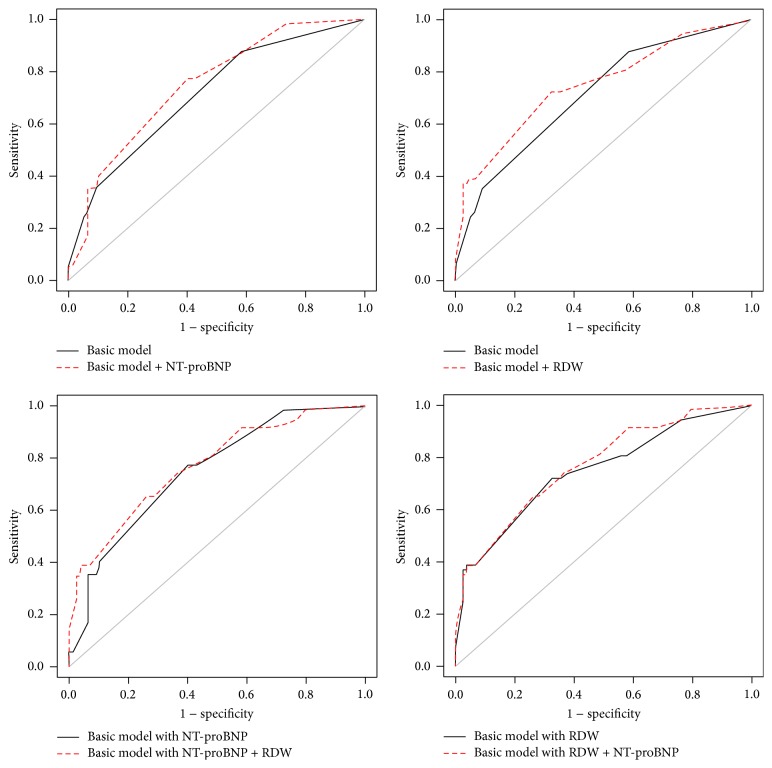
Increasing predictive capacity of the models. The *C*-statistics measures the overall AUCs of the prediction models and displays the overall predictive powers of the models in a more conservative fashion than Cox regression. AUC = area under the curve; RDW = red blood cell distribution width; NT-proBNP = N-terminal pro-B-type natriuretic peptide.

The *C*-statistics measures the overall areas under the curve (AUC) of the prediction models. Both the RDW [AUC = 0.71 to 0.75; *p* = 0.03] and NT-proBNP [AUC = 0.71 to 0.74; *p* = 0.04] improved the prediction statistically significantly beyond the basic model (Figure 4 and Table 4).

By adding RDW, we reached a reclassification improvement of 64% [NR I = 0.64 (0.33–0.95); *p* < 0.0001] and a discrimination development of a 3% [IDI = 0.03 (0.00–0.07), *p* = 0.01] over the NT-proBNP-adjusted basic model. Setting the NT-proBNP into the RDW-adjusted basic model did not result in such an improvement [NRI = 0.14 (−0.03–0.33); *p* = 0.11 and IDI = 0.01 (−0.01–0.03); *p* = 0.20], as demonstrated in Table 4. The multivariable model 2 suggested similar results (Table 5).

**Figure 5:**
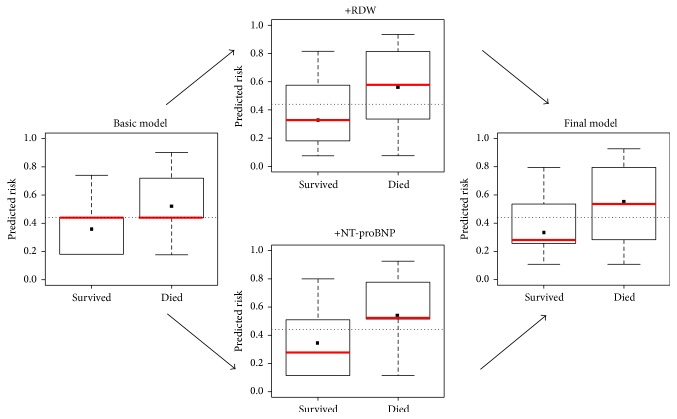
Improved discrimination of the prediction models. The improved discrimination capacity is graphically visualized by means of a box and whiskers diagram. The median (red line) of the mortality risk decreases in patients categorized as future survivors and increases in patients likely to die. The dotted line indicates the baseline risk. RDW = red blood cell distribution width; NT-proBNP = N-terminal pro-B-type natriuretic peptide.

The IDI = 0.03 indicates that 3% of the patients would have been better categorized by using the RDW in the final model: 4 of the 134 patients presented with low NT-proBNP but high RDW and died 5 years later.  Figure 5 displays this improved discrimination capacity; the median mortality risk [*p* = 0.42 (57/134)] decreases in patients categorized as future survivors (*p* = 0.22) and increases in patients likely to die (*p* = 0.55).

## 4. Discussion

### 4.1. Synopsis of Key Findings

We have shown that an increased RDW predicts the long-term mortality of chronic heart failure patients undergoing CRT, independently of the NT-proBNP level or other factors. The reclassification analyses revealed that the RDW has a higher risk stratification value than that of NT-proBNP in mortality prediction. Increased baseline RDW levels also predicted the lack of echocardiographic reverse remodeling, but NT-proBNP was superior from the aspect of CRT response prediction.

### 4.2. Possible Mechanisms and Explanations

Prediction models with RDW in chronic heart failure patients have been previously investigated [5–8], but the underlying mechanism remains unclear. Chronic heart failure is associated with systemic inflammation, oxidative stress, cytokine production, and neurohumoral activation, leading to enhanced catecholamine release and sympathetic activation [21]. Catecholamines downregulate renal erythropoietin production and cause consecutive anemia [22]. On the other hand, the sympathetic activation increases the cardiac output and stroke volume resulting in a volume redistribution that reduces the renal blood flow [22]. Furthermore, the upregulated plasma corticosteroids suppress the hematopoiesis in the bone marrow [23]. Development of the “cardiorenal syndrome” is characterized by anemia and a renal dysfunction. We therefore adjusted the serum creatinine data in the prediction models so as to reduce the possible influence of a renal dysfunction on the RDW values.

Very intensive clinical research is ongoing for biomarkers that predict the long-term survival of CRT patients, and biostatistics has a decisive role in these investigations [12]. The most commonly used method is Cox regression analysis, though it is of limited value in real clinical benefit assessments, as it overestimates the prediction if not controlled [24]. In contrast, the *C*-statistics, which investigates the overall AUCs of the models, is a rather conservative method and underestimates the benefit [24]. Novel reclassification approaches have therefore been developed, that is, NRI and IDI, to reveal the absolute discrimination improvement [20]. In our analysis we made use of well-calibrated prediction models, and the results of *C*-statistics, NRI, and IDI confirmed the results of the Cox regression analysis.

A recent systematic meta-analysis demonstrated that increased RDW levels are associated with a poor prognosis in patients with chronic heart failure [8] and also concluded that natriuretic peptides affect mortality, so that the prognostic value of the RDW might be overestimated without adjustment to NT-proBNP. In fact, our data reveal that the predictive value of NT-proBNP might be overestimated without adjustment to the RDW. It should be added that appropriately performed survival analyses are mandatory if a firm conclusion is to be reached.

To date, only three studies have evaluated the role of RDW in mortality prediction of CRT patients [14–16]. In a study on 217 patients, Rickard et al. demonstrated that the presence of an elevated RDW is associated with an increased mortality risk and less reverse left ventricular remodeling in patients undergoing CRT [15]. Celikyurt et al. showed that RDW fractions were increased significantly 6 months after CRT in the nonresponders among 66 patients but remained unaltered in the responders [14]. The investigation by Topaz et al. on 156 patients indicated that increased RDW levels even 6 months and 12 months after implantation are associated with poor mortality [16]. The baseline RDW fractions proved to be strong and independent predictor factors, but these studies did not investigate the influence of NT-proBNP on the final outcome. Our study confirms and extends these previous findings. The Cox regression results were validated with reclassification analyses, and the data demonstrated that the RDW outperforms NT-proBNP in long-term prediction. A possible explanation could be that while the natriuretic peptides reflect the actual filling status of the heart and determine the short-term outcome, the RDW mirrors the extent of systemic injury and consequently the long-term outcome. Further, the half-life of NT-proBNP is approximately 25 minutes, whereas the RDW is associated with the history of the previous 130 days (i.e., the lifespan of the red blood cells); the natriuretic peptides may therefore vary rapidly in response to current influences, while the RDW may remain unchanged [25, 26].

In our study patients with elevated RDW levels before CRT implantation were in worse NYHA functional class, had less LBBB pattern in the ECG with wider QRS, and used less angiotensin convertase inhibitor medications. They also tended to be older and to have diabetes more frequently. The RDW elevation is therefore most likely the consequence of the multiple comorbidities and disease progression, being a nonspecific biomarker. Nevertheless, reclassification analyses considered these confounders and RDW was still able to expand the risk prediction.

We established earlier that the ratio of the neutrophil leucocytes to the lymphocytes (the NL ratio) predicts the mortality in CRT independently of the NT-proBNP level [17]. We therefore propose that nonspecific biomarkers (such as the NL ratio or the RDW) might be considered in the clinical risk assessment scenarios. We in no way wish to underestimate the relevance of NT-proBNP measurements, but we would like to point out that a single, simple blood parameter could provide valuable additional prognostic information.

### 4.3. Strengths and Limitations

The main strength of this study is that it show for the first time the additional value of the RDW, a widely available biomarker in the risk assessment for CRT patients. The results clearly demonstrate the benefit of the RDW in risk prediction in a clinical setting. The investigation is limited by the relatively small sample size, which may have resulted in lower sensitivity and specificity. Moreover, the present results reported here derive from the analysis of an earlier published study by our group. All-cause mortality was taken as the end-point of the study and a distinction was not made between cardiovascular and noncardiovascular death due to low event numbers. The Teicholz method was used to compute left ventricular volumes instead of Simpson's method. Finally, the baseline characteristics of the two RDW groups differed; nevertheless the reclassification analyses considered these confounders.

The present investigation was obviously unable to include all relevant factors affecting the outcome in CRT, which might also bias the results. This analysis is mainly hypothesis-generating and the results should be regarded therefore as preliminary. Larger trials are needed to confirm the results.

## 5. Conclusions

Increased RDW levels predict the 5-year mortality of CRT patients independently of the NT-proBNP concentrations. Reclassification analysis revealed that the RDW might result in a better risk stratification than that with NT-proBNP and could lead to a better patient selection for CRT.

## Figures and Tables

**Table 1:** Baseline parameters as predictors of the 5-year mortality.

	Heart failure patients	Survived (*n* = 77)	Died (*n* = 57)	*p* value	Five-year mortality			
	(*n* = 134)				HR	95% CI of HR	Wald *χ* ^2^	*p* value
Clinical variables								
Age (years)	67 (60–73)	67 (60–71)	70 (62–74)	0.08	1.28	0.96–1.71	2.99	0.08
Male gender	110 (82)	59 (76)	51 (89)	0.05	2.06	0.88–4.81	2.82	0.09
BMI (kg/m^2^)	27 (24–30)	27 (25–30)	27 (23–29)	0.14	0.82	0.63–1.07	2.01	0.15
Ischemic	77 (57)	41 (53)	36 (63)	0.25	1.36	0.79–2.33	1.27	0.25
LBBB	110 (82)	70 (90)	40 (70)	0.002	0.35	0.20–0.63	12.52	<0.0001
CRT-D	22 (16)	14 (18)	8 (14)	0.52	0.72	0.34–1.54	0.68	0.40
Opt. lead position	98 (73)	56 (72)	42 (73)	0.90	1.00	0.55–1.80	0.00	1.00
QRS (msec)	163 (140–184)	164 (140–184)	163 (142–185)	0.82	1.05	0.81–1.36	0.14	0.70
LVEF (%)	28 (23–33)	28 (23–32)	26 (23–33)	0.90	0.96	0.73–1.27	0.05	0.82
LVESV (mL)	210 (153–276)	218 (158–276)	202 (140–259)	0.30	0.90	0.68–1.20	0.45	0.50
LVEDV (mL)	303 (250–361)	308 (250–381)	294 (242–341)	0.39	0.90	0.69–1.18	0.50	0.47
NYHA III, IV	115 (85)	63 (81)	52 (91)	0.12	2.07	0.82–5.19	2.42	0.12
Hypertension	70 (52)	42 (54)	32 (56)	0.85	1.04	0.61–1.75	0.02	0.88
Hyperlipidemia	32 (23)	16 (20)	16 (28)	0.32	1.20	0.67–2.14	0.39	0.53
Diabetes m.	48 (35)	26 (33)	24 (42)	0.32	1.51	0.89–2.56	2.36	0.12
ACEi/ARB	128 (95)	75 (97)	53 (92)	0.22	0.54	0.19–1.49	1.39	0.23
BB	119 (88)	73 (94)	47 (82)	0.02	0.34	0.17–0.69	9.11	0.003
MRI	93 (69)	57 (74)	37 (64)	0.25	0.71	0.41–1.23	1.46	0.22
Laboratory data								
RDW (%)	13.6 (13.0–14.6)	13.3 (12.9–14.0)	14.2 (13.5–15.2)	<0.0001	1.48	1.25–1.75	20.89	<0.0001
Hematocrit (%)	42 (38–45)	42 (39–45)	40 (36–43)	0.01	0.70	0.53–0.92	6.21	0.01
NT-proBNP (pg/mL)	2612 (1454–5101)	2188 (997–3567)	4025 (2086–6482)	<0.0001	1.43	1.19–1.73	14.45	<0.0001
Creatinine (*μ*mol/L)	109 (79–134)	93 (74–113)	116 (91–148)	0.0002	1.42	1.18–1.71	14.25	<0.0001

Data are expressed as medians with interquartile ranges for continuous variables and as event numbers with percentages for categorical variables. For the comparison of continuous data, we used the Mann-Whitney test, whereas the chi squared test was applied for the comparison of the categorical variables. The 5-year mortality was assessed by using univariate Cox regression analysis. The hazard ratios refer to the presence versus the absence in the case of categorical variables and a 1 standard deviation increase in the case of continuous variables. HR = hazard ratio; CI = confidence interval; *χ* ^2^ = Wald chi squared; BMI = body mass index; Ischemic = ischemic etiology of the heart failure; LBBB = left bundle branch block; CRT-D = cardiac resynchronization therapy with implantable cardioverter defibrillator; Opt. lead position = lateral or posterolateral position; LVEF = left ventricular ejection fraction; LVESV = left ventricular end systolic volume; LVEDV = left ventricular end diastolic volume; NYHA III, IV = New York Heart Association classification 3-4; ACEi/ARB = angiotensin convertase inhibitor/angiotensin receptor blocker; BB = beta-blocker; MRI = mineralocorticoid receptor inhibitor; NT-proBNP = N-terminal of the prohormone brain natriuretic peptide; RDW = red blood cell distribution width.

**Table 2:** Baseline parameters as predictors of the 6-month reverse remodeling.

	Responders (*N* = 61)	Nonresponders (*N* = 62)	*p* value	Lack of reverse remodeling			
				OR	95% CI of OR	Wald *χ* ^2^	*p* value
Clinical variables							
Age (years)	65 (56–70)	68 (63–75)	0.003	1.84	1.22–2.78	8.51	0.004
Male gender	47 (77)	54 (87)	0.14	2.01	0.77–5.21	2.06	0.15
BMI (kg/m^2^)	27 (25–30)	27 (24–30)	0.68	0.89	0.62–1.28	0.37	0.53
Ischemic	33 (54)	41 (66)	0.17	1.65	0.80–3.43	1.84	0.17
LBBB	54 (88)	49 (79)	0.15	0.48	0.18–1.32	1.98	0.15
CRT-D	11 (18)	8 (12)	0.43	0.67	0.25–1.81	0.61	0.43
Opt. lead position	44 (72)	44 (70)	0.88	0.94	0.43–2.06	0.02	0.88
QRS (msec)	160 (160–180)	160 (140–181)	0.62	0.89	0.62–1.28	0.35	0.55
LVEF (%)	27 (22–31)	27 (23–33)	0.63	1.12	0.78–1.58	0.40	0.52
LVESV (mL)	234 (173–276)	188 (137–238)	0.01	0.63	0.42–0.94	5.01	0.01
LVEDV (mL)	331 (263–386)	267 (234–336)	0.006	0.62	0.42–0.91	5.91	0.01
NYHA III. IV	47 (77)	58 (93)	0.01	4.31	1.33–13.99	5.94	0.01
Hypertension	32 (52)	34 (54)	0.79	1.10	0.54–2.26	0.70	0.79
Hyperlipidemia	12 (19)	19 (30)	0.16	1.80	0.78–4.14	1.93	0.16
Diabetes m.	20 (32)	24 (38)	0.49	1.29	0.61–2.71	0.46	0.49
ACEi/ARB	58 (95)	60 (96)	0.63	1.55	0.25–9.62	0.22	0.63
BB	58 (95)	56 (90)	0.19	0.40	0.10–1.65	1.58	0.20
MRI	49 (80)	37 (59)	0.01	0.36	0.16–0.81	6.03	0.01
Laboratory data							
RDW (%)	13.4 (12.8–14.1)	13.9 (13.0–14.9)	0.01	1.52	1.01–2.29	4.07	0.04
Hematocrit (%)	42 (39–45)	40 (36–44)	0.06	0.70	0.49–1.02	3.35	0.06
NT-proBNP (pg/mL)	2277 (987–3627)	3126 (1665–6231)	0.002	2.00	1.19–3.38	6.88	0.009
Creatinine (*μ*mol/L)	96 (73–131)	113 (82–143)	0.03	1.56	1.04–2.34	4.75	0.02

Data are expressed as medians with interquartile ranges for continuous variables and as event numbers with percentages for categorical variables. For the comparison of continuous data, we used the Mann-Whitney test, whereas the chi squared test was applied for the comparison of the categorical variables. Reverse remodeling was defined as a relative decrease of at least 15% in the LVESV 6 months after CRT implantation without death. The lack of reverse remodeling was tested by using univariate logistic regression analyses. The odds ratios refer to the presence versus the absence in the case of categorical variables and a 1 standard deviation increase in the case of continuous variables. OR = odds ratio; CI = confidence interval; *χ* ^2^ = Wald chi squared; BMI = body mass index; Ischemic = ischemic etiology of the heart failure; LBBB = left bundle branch block; CRT-D = cardiac resynchronization therapy with implantable cardioverter defibrillator; Opt. lead position = lateral or posterolateral position; LVEF = left ventricular ejection fraction; LVESV = left ventricular end systolic volume; LVEDV = left ventricular end diastolic volume; NYHA III, IV = New York Heart Association classification 3-4; ACEi/ARB = angiotensin convertase inhibitor/angiotensin receptor blocker; BB = beta-blocker; MRI = mineralocorticoid receptor inhibitor; NT-proBNP = N-terminal of the prohormone brain natriuretic peptide; RDW = red blood cell distribution width.

**Table 3:** Differences in baseline characteristics in patients with increased or decreased RDW levels.

	RDW < 13.35%	RDW > 13.35%	*p* value
	(*n* = 53)	(*n* = 81)	
Clinical variables			
Age (years)	67 (59–71)	68 (62–74)	0.08
Male gender	43 (81)	67 (82)	0.81
BMI (kg/m^2^)	27 (24–30)	27 (23–29)	0.40
Ischemic	30 (56)	47 (58)	0.87
LBBB	49 (92)	61 (75)	0.005
CRT-D	11 (20)	11 (13)	0.27
Opt. lead position	41 (77)	57 (70)	0.37
QRS (msec)	155 (134–180)	164 (146–189)	0.04
LVEF (%)	28 (24–33)	25 (23–32)	0.43
LVESV (mL)	202 (147–276)	214 (153–267)	0.71
LVEDV (mL)	285 (234–341)	312 (250–361)	0.29
NYHA III. IV	40 (75)	75 (92)	0.005
Hypertension	29 (54)	45 (55)	0.92
Hyperlipidemia	11 (20)	21 (25)	0.49
Diabetes m.	15 (28)	35 (43)	0.08
ACEi/ARB	53 (100)	75 (92)	0.04
BB	49 (92)	71 (87)	0.37
MRI	37 (69)	57 (70)	0.94
Laboratory data			
Hematocrit (%)	43 (39–45)	41 (37–43)	0.11
NT-proBNP (pg/mL)	1817 (691–3086)	3581 (2007–6232)	<0.0001
Creatinine (*μ*mol/L)	92 (74–121)	112 (85–135)	0.007

Data are expressed as medians with interquartile ranges for continuous variables and as event numbers with percentages for categorical variables. For the comparison of continuous data, we used the Mann-Whitney test, whereas the chi squared test was applied for the comparison of the categorical variables. BMI = body mass index; Ischemic = ischemic etiology of the heart failure; LBBB = left bundle branch block; CRT-D = cardiac resynchronization therapy with implantable cardioverter defibrillator; Opt. lead position = lateral or posterolateral position; LVEF = left ventricular ejection fraction; LVESV = left ventricular end systolic volume; LVEDV = left ventricular end diastolic volume; NYHA III, IV = New York Heart Association classification 3-4; ACEi/ARB = angiotensin convertase inhibitor/angiotensin receptor blocker; BB = beta-blocker; MRI = mineralocorticoid receptor inhibitor; NT-proBNP = N-terminal of the prohormone brain natriuretic peptide; RDW = red blood cell distribution width.

**Table 4:** Multivariable Cox regression analysis of the 5-year mortality with validation, calibration, and reclassification.

	Basic model with RDW + NT-proBNP	Basic model + RDW	Basic model	Basic model + NT-proBNP	Basic model with NT-proBNP + RDW
Cox regression					
HR of RDW	2.49 (1.27–4.86)	2.81 (1.45–5.44)			2.49 (1.27–4.86)
*p* (RDW)	0.008	0.002			0.008
HR of NT-proBNP	1.18 (0.93–3.51)			2.19 (1.13–4.23)	1.18 (0.93–3.51)
*p* (NT-proBNP)	0.07			0.01	0.07
Validation					
Overall *χ* ^2^	42.62	40.47	**29.86**	35.32	42.62
*p* (overall)	<0.0001	<0.0001	**<0.0001**	<0.0001	<0.0001
*p* (changes)	0.06	0.001		0.01	0.004
Calibration				
HL test *χ* ^2^	8.26	6.72	**0.48**	4.01	8.26
*p* (HL test)	0.40	0.56	**0.99**	0.85	0.40
Performance					
Brier score	0.18	0.19	**0.20**	0.19	0.18
Nagelkerke's *R* ^2^	0.30	0.28	**0.21**	0.26	0.30
Reclassification					
*C*-statistics	0.76 (0.69–0.84)	0.75 (0.66–0.83)	**0.71 (0.63–0.79)**	0.74 (0.66–0.82)	0.76 (0.69–0.84)
*p* (*C*-statistics)	0.80	0.03		0.04	0.39
NRI (95% CI)	0.14 (–0.03–0.33)	0.64 (0.33–0.95)		0.54 (0.23–0.84)	0.64 (0.33–0.95)
*p* (NRI)	0.11	<0.0001		0.0005	<0.0001
IDI (95% CI)	0.01 (–0.00–0.03)	0.05 (0.01–0.09)		0.03 (0.00–0.06)	0.03 (0.00–0.07)
*p* (IDI)	0.20	0.003		0.04	0.01

The basic multivariable Cox regression model included left bundle branch block, beta-blocker therapy, and creatinine > 88.5 *μ*mol/L. The RDW > 13.35% and NT-proBNP > 1975 pg/mL were adjusted separately to the basic model in a forward stepwise manner. The final common model included all variables. HR = hazard ratio; *χ* ^2^ = chi squared; HL test = Hosmer-Lemeshow test; 95% CI = 95% confidence interval; *C*-statistics = overall areas under the curve, assessed by the DeLong test; NRI = net reclassification improvement; IDI = integrated discrimination improvement. NT-proBNP = N-terminal pro-B-type natriuretic peptide; RDW = red blood cell distribution width.

**Table 5:** Multivariable Cox regression analysis 2 of the 5-year survival with validation, calibration, and reclassification.

	Basic model with RDW + NT-proBNP	Basic model + RDW	Basic model	Basic model + NT-proBNP	Basic model with NT-proBNP + RDW
Cox regression					
HR of RDW	2.42 (1.22–4.76)	2.07 (1.38–5.29)			2.42 (1.22–4.76)
*p* (RDW)	0.01	0.004			0.01
HR of NT-proBNP	1.88 (0.95–3.69)			2.21 (1.13–4.33)	1.88 (0.95–3.69)
*p* (NT-proBNP)	0.06			0.02	0.06
Validation					
Overall *χ* ^2^	46.08	43.52	**33.90**	39.10	46.08
*p* (overall)	<0.0001	<0.0001	**<0.0001**	<0.0001	<0.0001
*p* (changes)	0.05	0.002		0.01	0.006
Calibration				
HL test *χ* ^2^	4.65	1.78	**11.75**	4.71	4.65
*p* (HL test)	0.79	0.98	**0.16**	0.78	0.79
Performance					
Brier score	0.18	0.18	**0.19**	0.19	0.18
Nagelkerke's *R* ^2^	0.34	0.32	**0.25**	0.29	0.34
Reclassification				
*C*-statistics	0.80 (0.72–0.87)	0.78 (0.70–0.85)	**0.74 (0.66–0.82) **	0.76 (0.68–0.84)	0.80 (0.72–0.87)
*p*(*C*-statistics)	0.39	0.20		0.29	0.11
NRI (95% CI)	0.24 (–0.03–0.34)	0.64 (0.33–0.95)		0.54 (0.23–0.84)	0.64 (0.33–0.95)
*p* (NRI)	0.19	<0.0001		0.0005	<0.0001
IDI (95% CI)	0.01 (–0.01–0.03)	0.05 (0.01–0.09)		0.03 (−0.00–0.06)	0.04 (0.01–0.07)
*p* (IDI)	0.18	0.004		0.05	0.01

The basic multivariable Cox regression model included age, male gender, NYHA class III/IV, LBBB, beta-blocker therapy, diabetes mellitus, and creatinine > 88.5 *μ*mol/L. The RDW > 13.35% and NT-proBNP > 1975 pg/mL were adjusted separately to the basic model in a forward stepwise manner. The final common model included all variables. HR = hazard ratio; *χ* ^2^ = chi squared; HL test = Hosmer-Lemeshow test; 95% CI = 95% confidence interval; *C*-statistics = overall areas under the curve, assessed by the DeLong test; NRI = net reclassification improvement; IDI = integrated discrimination improvement. NT-proBNP = N-terminal pro-B-type natriuretic peptide; RDW = red blood cell distribution width.
